# Self-Supported Branched Poly(ethylenimine) Monoliths from Inverse Template 3D Printing for Direct Air Capture

**DOI:** 10.1021/acsami.4c20617

**Published:** 2025-02-11

**Authors:** Pavithra Narayanan, Seo-Yul Kim, Dema Alhazmi, Christopher W. Jones, Ryan P. Lively

**Affiliations:** School of Chemical and Biomolecular Engineering, Georgia Institute of Technology,Atlanta, Georgia 30332, United States

**Keywords:** CO_2_ capture, direct air capture, monoliths, 3D printing, amine adsorbent, ice templating

## Abstract

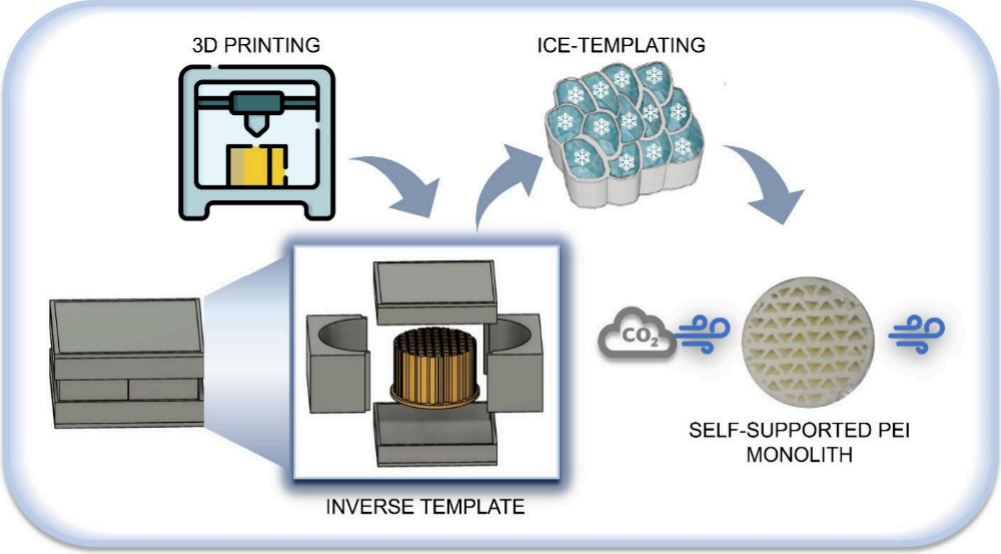

3D-printed inverse templates are combined with ice templating to develop self-supported branched poly(ethylenimine) monoliths with regular channels of varying channel density and ordered macropores. A maximum uptake of 0.96 mmol of CO_2_/g of monolith from ambient air containing 45.5% RH is achieved from dynamic breakthrough experiments, which is a 31% increase compared to the CO_2_ uptake from adsorption under dry conditions for the same duration. The breakthrough experiments show characteristics of internal mass-transfer limitations. The cyclic dynamic breakthrough experiments indicate stable operation without significant loss in CO_2_ uptake across eight cycles. Moreover, the self-supported monolith shows minimal loss in adsorption capacity (7.7%) upon exposure to air containing 21% oxygen at 110 °C, in comparison to a conventional sorbent consisting of poly(ethylenimine) impregnated on Al_2_O_3_ (18.9%). The monoliths exhibit good mechanical stability, contributed by elastic deformation, corresponding to up to 74% strain and lower pressure drop compared to many existing monoliths in the literature.

## Introduction

Global temperatures have been increasing since the preindustrial period. Greenhouse gas emissions contribute significantly to global warming, and 60% of the effect is attributed to CO_2_.^1^ Several studies have shown that anthropogenic climate change is irreversible on millennial time scales, even if further CO_2_ emissions are completely ceased.^2−5^ This “point of no return” has necessitated aggressive negative emission technologies to complement point-source carbon capture in combating the current effects of climate change within time scales relevant to humankind. In this context, amines are promising candidates for inclusion in sorbents for direct air capture (DAC) due to their strong chemisorption interaction with CO_2_. These materials have demonstrated promising CO_2_ uptake results under dry and humid conditions, making them suitable for capturing CO_2_ from very dilute sources.^6−13^

Much of the literature for CO_2_ capture has historically focused on sorbent development, understanding the interactions between the sorbent material and CO_2_, and tuning the sorbent chemistry for better CO_2_ uptake and sorbent stability.^14−23^ While these are important aspects that determine the CO_2_ uptake, kinetics, and thermodynamic limits of a capture process, it is also essential to consider the form and macroscopic structure of the sorbent. These affect the external gas transport properties and, when coupled with the operating parameters, determine the residence time for the contact of gas molecules with the sorbent and pressure drop, which are relevant in large-scale deployment of the process and determining energy and operating cost requirements. The ultradilute concentration of CO_2_ in ambient air means that a larger volume of air would have to be processed to capture the same amount of CO_2_ as from a comparatively more concentrated source such as flue gas. The high throughput could result in a high pressure drop, that can potentially be minimized by structuring the sorbent. This requirement of a reduced pressure drop further emphasizes the need to evaluate sorbents in their macroscopic form.

Solid sorbents can exist in different configurations such as powders,^7,9,18,24−27^ beads,^28^ pellets,^29−31^ fibers,^32−38^ laminates,^39,40^ and monoliths.^11,12,41−44^ Among them, monoliths are known to have the lowest pressure drop for a wide range of inlet feed velocities.^45^ Straight channel monoliths, used in catalytic converters for several decades, offer significant potential in helping fulfill the gas transport needs for CO_2_ capture.^11,12,46,47^ These monoliths also offer heat integration opportunities via water channels separate from the gas transport channels.^48^

A wide range of amine-functionalized monoliths have been explored in the literature for CO_2_ capture. For example, Sakwa-Novak et al. impregnated poly(ethylenimine) (PEI) on extruded alumina monolithic honeycomb structures to obtain a stable cyclic CO_2_ uptake of 0.7 mmol/g of monolith from simulated ambient air.^42^ Similar monoliths functionalized with triamine by Grossmann et al. exhibit an uptake of 0.4 mmol of CO_2_/g of sorbent.^49^ Darunte et al. developed amine-functionalized Mg_2_(dobpdc), a metal–organic framework (MOF), by forming a MOF film on α-Al_2_O_3_ wash-coated cordierite monoliths and functionalizing the same with amine groups.^12^ A CO_2_ uptake from simulated flue gas of 2.37 mmol/g of sorbent was achieved. PEI-loaded hierarchically porous silica monoliths by Guo et al. showed a CO_2_ uptake of 1 mmol/g of sorbent from a similar model flue gas.^50^

While there have been continuous improvements leading to better understanding of amine-functionalized monoliths over time, most of these monoliths are composed of inactive support that does not contribute directly to the CO_2_ uptake. The support material acts as a dead weight, contributing to the energy penalty during thermal regeneration. Moreover, there is a limit on the number of amines, the active component, that can be loaded onto the support without forming amine aggregates.^51^ Self-supported amine sorbents can potentially address these drawbacks. To this end, in earlier research preceding this study, self-supported amine scaffolds were synthesized using ice templating.^52−54^ Ice templating involves the nucleation and growth of ice crystals from an aqueous solution at a subambient temperature, with simultaneous phase inversion of other components in an aqueous phase. This phase inversion is induced by external stimuli such as a chemical reaction.^55,56^ The ice crystals act as a template during this process, producing porosity when thawed.^57^ The anisotropy of the structures and the interconnected ordered porosity formed using ice templating, along with the versatility of the process and ability to tune the morphology of the synthesized material using factors such as freezing direction, temperature, concentration of constituents and presence of an additive have attracted interest, making this a promising method for developing materials with applications in gas transport.^58^ Dong et al. developed aerogels consisting of quaternized cellulose and ion-exchange resin using ice templating for DAC with moisture swing adsorption. A maximum CO_2_ uptake of 1.17 mmol/g of sorbent was demonstrated at high nitrogen loading, with a working capacity of 0.5 mmol of CO_2_/g of sorbent.^59^ However, most sorbents synthesized using the templating method are small cylinders or cuboids without channels that cannot be used as they are, due to extreme mass-transfer limitations and high pressure drops.^50,60−65^ Hence, all performance testing included in our prior work with ice templating was conducted on small sections of the sorbent or by cryomilling the sorbent and treating them as pelletized powders. Conventional monolith fabrication methods such as extrusion are not compatible with ice templating. Extruding at subzero temperatures necessitated by ice templating would likely result in structural collapse due to incomplete phase inversion, making it challenging to apply the method for developing self-supported porous monoliths with well-defined channels. A technique utilizing the merits of ice templating and simultaneously fabricating monoliths at scale with tunable macroscopic structures that can be used as synthesized in DAC will help enable the translation of the performance of the sorbents as seen in initial testing into larger-scale deployments. On the other hand, 3D printing, or additive manufacturing, has gained traction as an emerging cost-effective and facile technology for fabricating adsorption contactors.^66−71^ This process typically involves layer-by-layer printing of either molten commercially available filament-fused deposition modeling (FDM) or custom-made “inks” that can undergo instantaneous phase change in response to stimuli–direct ink writing (DIW), the latter of which has been used in the fabrication of monolithic sorbents.^66−69,71,72^ Recent developments in the field have enabled the fabrication of 3D structures with feature sizes in the microscopic length scale with simultaneous design complexity in the macroscopic length scale, a notable limitation of the conventional extrusion method.

A combination of 3D printing and ice templating can bring together the merits of the two techniques: easy fabrication of complex macroscopic structures from 3D printing and ordered, aligned, and interconnected porosity from ice templating. While ice templating requires subzero temperatures for the nucleation and growth of ice crystals, such an environment results in slow reaction kinetics for the chemical reaction that causes phase change. These slow reaction kinetics are not conducive in DIW because this technique requires a layer to undergo instantaneous phase change before printing the next layer. However, 3D printing, sequentially followed by ice templating, can overcome this limitation and develop materials with multiscale porosity and well-defined macrochannels. This approach has been utilized previously to develop structured materials for applications in bioceramics^73−75^ and batteries^76^ but remains underexplored, with no known study of gas transport in such materials.

In this work, the sequential use of 3D printing and ice templating is demonstrated as a method for fabricating monoliths for gas transport and adsorption. Self-supported, branched PEI monoliths with defined channel dimensions were synthesized using a combination of ice templating and inverse template 3D printing for the first time in CO_2_ capture applications. The monoliths were evaluated using ambient room air, emphasizing practical considerations, including quantification of pressure drop, mechanical properties, and the effect of oxidation.

## Materials and Methods

Branched poly(ethylenimine) (b-PEI 800, *M*_w_ = 800, and *M*_n_ = 600; referred to in this work as PEI800), 50 wt % branched poly(ethylenimine) [b-PEI, *M*_w_ = 750000 by light scattering (LS) and *M*_n_ = 60000 by gel permeation chromatography (GPC)] in aqueous solution (referred to in this work as PEI60k), poly(ethylene glycol) diglycidyl ether (PEGDGE, *M*_n_ = 500), and methanol (99.8% ACS grade) were purchased from Sigma-Aldrich and used as received. Catalox HTa γ-Al_2_O_3_ was obtained from Sasol. Washing solvents such as methanol (CH_3_OH, >99.8%), hexane (C_6_H_14_, >95%), and acetone [(CH_3_)_2_CO, >99.5%] were purchased from VWR and used directly. Nitrogen (UHP 5.0 grade), helium (UHP 5.0 grade), ultra zero grade air, and specialty gas mixtures of 400 ppm of CO_2_ in helium and nitrogen were purchased from Airgas Inc. Fused Material’s water-soluble poly(vinyl alcohol) (PVA) filaments and Sunlu’s poly(lactic acid) (PLA) filaments, both 1.75 mm thick, were purchased from Amazon and used after drying overnight at 50 °C to remove moisture. The ambient air from the room was used for humid adsorption experiments after monitoring the temperature and relative humidity.

### Synthesis of Alumina-Supported Amines

Supported amine sorbents have been organized into three classes: amines physically impregnated into the pores of a support, amines covalently bonded to the support, and amines polymerized in situ resulting in polyamines tethered to the support.^77^ As an example of a class 1 amine sorbent, 35 wt % PEI impregnated on γ-Al_2_O_3_, denoted as PEI@Al_2_O_3_, was synthesized. This sorbent is referred to as the benchmark material. Low-molecular-weight PEI800 was used for the same. PEI was impregnated on γ-Al_2_O_3_ by a wet impregnation method.^78,79^ In short, 1 g of γ-Al_2_O_3_ dried in a convection oven at 105 °C for at least 48 h was dispersed in 15 mL of methanol. Separately, 540 mg of PEI800 was dissolved in 20 mL of methanol. The two mixtures were stirred separately at room temperature at 300 rpm for at least 1 h before mixing dropwise by adding the PEI/methanol mixture to the γ-Al_2_O_3_ dispersion. The mixture was stirred for 24 h at room temperature. Methanol was removed in a rotary evaporator at 164 Torr, with a water bath at 50 °C. The sample was then dried at 100 °C overnight under a high vacuum of 12 mTorr. The performance of the self-supported PEI monolith synthesized was compared against this benchmark material.

### Design and 3D Printing of Templates

**Figure 1:**
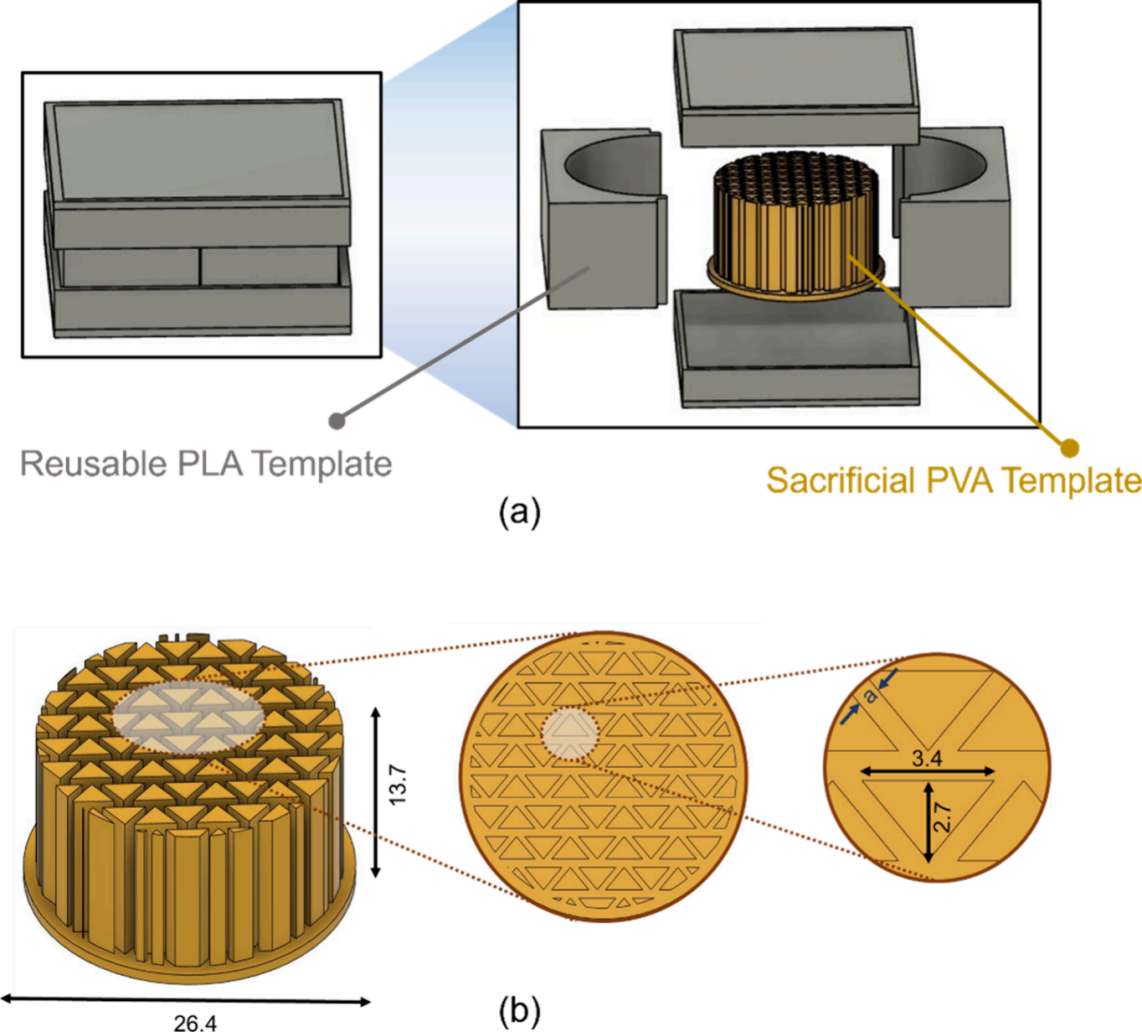
(a) Sample CAD image of the template components designed in *Autodesk Fusion 360*, depicting their assembly. (b) Enlarged view of the PVA template. The spacing between 3D-printed pillars, i.e., the wall thickness of the monolith, is denoted by “a”. All dimensions are in millimeters.

A five-piece template consisting of four reusable components made of PLA and one sacrificial piece made of PVA were 3D-printed using Bambu X1 Carbon, an FDM 3D printer. First, a computer-aided design (CAD) of the templates was developed in *Autodesk Fusion 360* software. The sacrificial PVA component was designed to be an inverse of the desired triangular straight channels in the monolith structure. This approach was described previously by Kim et al. for the fabrication of sorbent-loaded cellulose acetate contactors.^80^ Triangular channels were chosen due to their superior structural integrity, stress distribution, and heat and mass transfer compared to other straight channel geometries. The triangular lattice ensures uniform stress distribution and enhances mechanical stability, while the higher surface area-to-volume ratio in triangular channels for a given wall thickness and packing fraction promote more efficient heat and mass transfer than square channels.^81,82^ Three different PVA templates were designed to obtain monoliths of different wall thicknesses and, thereby, different channel densities. The maximum attainable channel density is dependent on the resolution of the printer. A sample CAD drawing of the template assembly is depicted in Figure 1. The CAD design is then sliced in *Bambu v1.7.7.89*, an open-source software, to obtain a g-code. Slicing is the process of converting the CAD image into a format that is readable by the 3D printer. The g-code also includes information on how each design layer will be printed on the print bed and the temperature of the print bed and extrusion nozzle during the printing process. The print path for the layers and print speed were optimized to allow sufficient time for each layer to cool down before printing the next layer over the same. This consideration is crucial because the template consists of thin and tall triangular pillars that could collapse otherwise.

The temperature of the bed and the nozzle depend on the type of filament printed. The bed’s temperature influences the adhesion between the bed surface and the first printed layer, and it indirectly impacts layer-to-layer adhesion by increasing the air temperature within the enclosure. The nozzle’s temperature affects the filament’s fluidity during printing and is also an important factor positively affecting the layer-to-layer adhesion. For PLA, the temperature of the bed and nozzle were 70 and 210 °C, respectively, while the same for PVA were 70 and 205 °C, respectively. The slicing parameters were optimized for the used filaments. It is essential to choose the right temperature for extrusion, especially for PVA, because the filament was found to clog the extrusion nozzle at temperatures over 215 °C, potentially from unsaturation, chain scission, and cross-linking of PVA.^83^ The slicing parameters were varied, and trial prints were conducted. The optimal conditions used in this work for the PLA and PVA parts are shown in Figures S1 and S2, respectively. The components thus printed were assembled, as shown in Figure 1, and used as a reaction vessel for the cross-linking reaction of PEI60k.

### Synthesis of PEI Monoliths

The macroscopic structure of a sorbent affects the residence time for the contact of gas molecules with the sorbent. Self-supported b-PEI monoliths of varying size and channel density were synthesized by ice templating. The reaction in the ice-templating process was the same as that described in previous works and was modeled from the work of Chatterjee et al.^52−54,63^ Unlike in the previous studies, the cross-linking was performed in the custom-made template described in the previous section, instead of a vial or plastic tube. This setup allowed the modification of the monolith size, channel size, wall thickness, and, thereby, the channel density. Thus, the monolith was scaled up by a factor of ∼10 by mass, depending on the channel density. Briefly, a stock solution containing 9.09 wt % PEI60k was prepared by diluting a 50 wt % PEI60k solution obtained from Sigma-Aldrich in deionized (DI) water. Then, a desired amount of the above PEI stock solution was added to PEGDGE in a vial and mixed in a vortex mixer at 500 rpm for 10 s. The volume of the PEI stock solution varied depending on the desired channel density of the monolith, and the volumetric ratio of PEI60k and PEGDGE was maintained at 8:1. This ratio was found to be promising based on a prior study.^52^ The mixture was quickly poured into the template, sealed with parafilm, and completely immersed in a liquid nitrogen bath at −196 °C for 2 min before being placed in a freezer at −10 °C for 2 days. The template with monolith was thawed at room temperature, and the outer PLA shells were removed. The inner sacrificial PVA template and the cross-linked PEI monolith were immersed in DI water and left undisturbed for 2 days, with the DI water replaced once in between. The sacrificial PVA template was completely dissolved in DI water, resulting in a self-supported b-PEI monolith, which was sequentially washed in methanol and hexane before drying in a vacuum oven (∼30 in Hg) overnight at 40 °C. The sequential solvent exchange from water to methanol and then to hexane (from a solvent with higher surface tension to that with lower surface tension) prevents or limits the collapse of pores in these flexible sorbents. The monoliths thus prepared were named PEI_196_1X, PEI_196_2X, or PEI_196_3X, depending on the channel density. The theoretical channel densities of the monoliths were 79, 60, and 48 channels per square inch (CPSI) respectively. A previous study demonstrated improved CO_2_ uptake performance in the presence of Sasol γ-Al_2_O_3_ as an additive in the self-supported PEI matrix.^54^ Hence, Al_2_O_3_@PEI_196_2X, another monolith prepared in the same method but with an addition of Sasol γ-Al_2_O_3_ (6.5 wt % in the final monolith) in the reaction mixture was used for the oxidation studies described in the Characterization section.

### Characterization

#### Micro-Computed X-ray Tomography (Micro-CT) Imaging

The 3D structure of the monoliths and templates was visualized using micro-CT imaging. Images were recorded with a layer height of 34 μm using μCT 50 micro-CT scanner by SCANCO Medical AG, Switzerland. Mimics 24, an interactive image control system, and its subsidiary software, *Materialise Mimics*, were used to construct a 3D model from the obtained layers. *Autodesk Fusion 360*, a CAD software, was used to perform geometrical analysis on the 3D models.

#### Scanning Electron Microscopy (SEM)

SEM was used to study the channel morphology and understand the pore distribution in the monolith. A thin horizontal slice of the monoliths, PEI_196_1X, PEI_196_2X, and PEI_196_3X, was mounted on an SEM stub using carbon tape. The samples were gold-coated with a sputterer (Hummer VI) for 30 s. This step prevented charging and the resulting anomalous contrast in nonconductive samples. A staging distance between 8 and 11 mm was maintained. Images were captured at an accelerating voltage of 3 kV in Thermo Fischer Helios 5CX FIB-SEM. The obtained images were analyzed using *ImageJ v1.53e*, an open-source software.

### CO_2_ Adsorption

Dynamic CO_2_ adsorption breakthrough studies from dry 400 ppm of CO_2_ balanced by N_2_ and indoor air containing humidity were performed in a custom-built fixed bed. A schematic of the setup is shown in Figure S3, and its operation is described in detail in Section S2. The monoliths were degassed at 100 °C under N_2_ until no CO_2_ or water was detected by the LiCOR downstream. The flow rate of N_2_ was 500 sccm. For CO_2_ adsorption, the feed consisted of either 400 ppm of CO_2_/N_2_ (dry) from a custom-ordered cylinder or indoor air (448 ± 7 ppm, 18.5 ± 0.32 °C, and 56.6 ± 8.7% RH) flowing at 500 sccm, pushed into the fixed bed maintained at about 25 °C using a blower and flow control setup. The adsorption was carried out until the ratio of the concentration of CO_2_ in the outlet stream and that in the feed stream was 0.98. For the experiments involving indoor air, the adsorption step was preceded by a presaturation step where humid N_2_ (85% RH) flowed through the monolith at a flow rate of 200 sccm. This step was done to deconvolute any competitive effects of H_2_O in CO_2_ adsorption. Similar to the activation step explained above, a desorption step was performed to regenerate the monolith.

### Pressure-Drop Measurement

The pressure drop across the PEI_196_1X, PEI_196_2X, and PEI_196_3X monoliths was measured as a function of superficial velocity. A custom-built lab-scale setup, shown in Figure S4, was used for this purpose. The module for this experiment was the same as the one used in CO_2_ uptake measurements, with an additional pressure gauge measuring the pressure difference across the two ends of the module while flowing nitrogen at varying inlet gas velocity. The temperature of the module was maintained at 25 °C. The pressure drop generated by the empty module was also measured at the corresponding inlet gas velocities and subtracted from that measured with the monolith.

### Oxidation Studies

The relative loss in CO_2_ uptake of the sorbents was used to track the oxidative degradation of the monolithic sorbents. Accelerated oxidation was performed on the sorbents by exposing them to 21% O_2_/N_2_ dry oxidative conditions at 110 °C for 3 h in a TA Instruments Q550 thermogravimetric analyzer. The sorbents were activated before the oxidation step by purging with 90 mL/min of N_2_ at 100 °C. The CO_2_ uptake of the sorbents was measured gravimetrically (TA Instruments Q500) before and after exposure to the oxygen-containing environment. In an adsorption experiment, 400 ppm of CO_2_/N_2_ was flowed through 15 mg of the sorbent at 25 °C for 3 h. The adsorption was preceded by activation, following the same procedure followed before exposing the sorbents to an oxidative environment. A temperature of 110 °C was chosen as the oxidation temperature because that is the typically used desorption temperature of PEI impregnated on Al_2_O_3_ sorbents and the highest temperature that a DAC plant with such sorbents undergoing temperature swing adsorption would experience. PEI impregnated on Al_2_O_3_ with a 35 wt % PEI loading (PEI@Al_2_O_3_), self-supported PEI monolith (PEI_196_2X), and self-supported PEI monolith with 6.5 wt % Al_2_O_3_ as an additive (Al_2_O_3_@PEI_196_2X) were used for the oxidation studies. It should be noted that the sorbents are expected to be exposed to oxidative conditions at such high temperatures only in accidental cases and process upsets.

### Compression Experiments

Compression tests were performed on the Mark-10 ESM303 mechanical tester. A 250 N rated force gauge was lowered on the monolith from its surface at a 10 mm/min loading rate until fracture.

## Results and Discussion

### Macroscopic Structure of Monoliths

**Figure 2:**
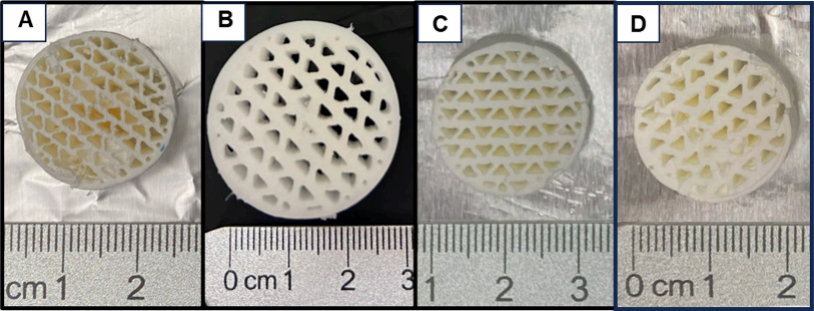
Top view of the ice-templated self-supported PEI monoliths (a) dried PEI_196_1X (b) wet PEI_196_2X (c) dried PEI_196_2X, and (d) dried PEI_196_3X.

Self-supported PEI monoliths with three different channel sizes were fabricated using ice templating, and the samples obtained are shown in Figure 2. The cross-linked network of the sorbent remains swollen during the initial steps of PVA dissolution and solvent exchange. An example of this swollen state is depicted in Figure 2b. The monoliths experience shrinkage upon drying, which is quantified as discussed later in this section. It should be noted that while the monoliths are structurally stable when dry, they are fragile in the swollen state, which may pose challenges in handling upon scale-up.

**Figure 3:**
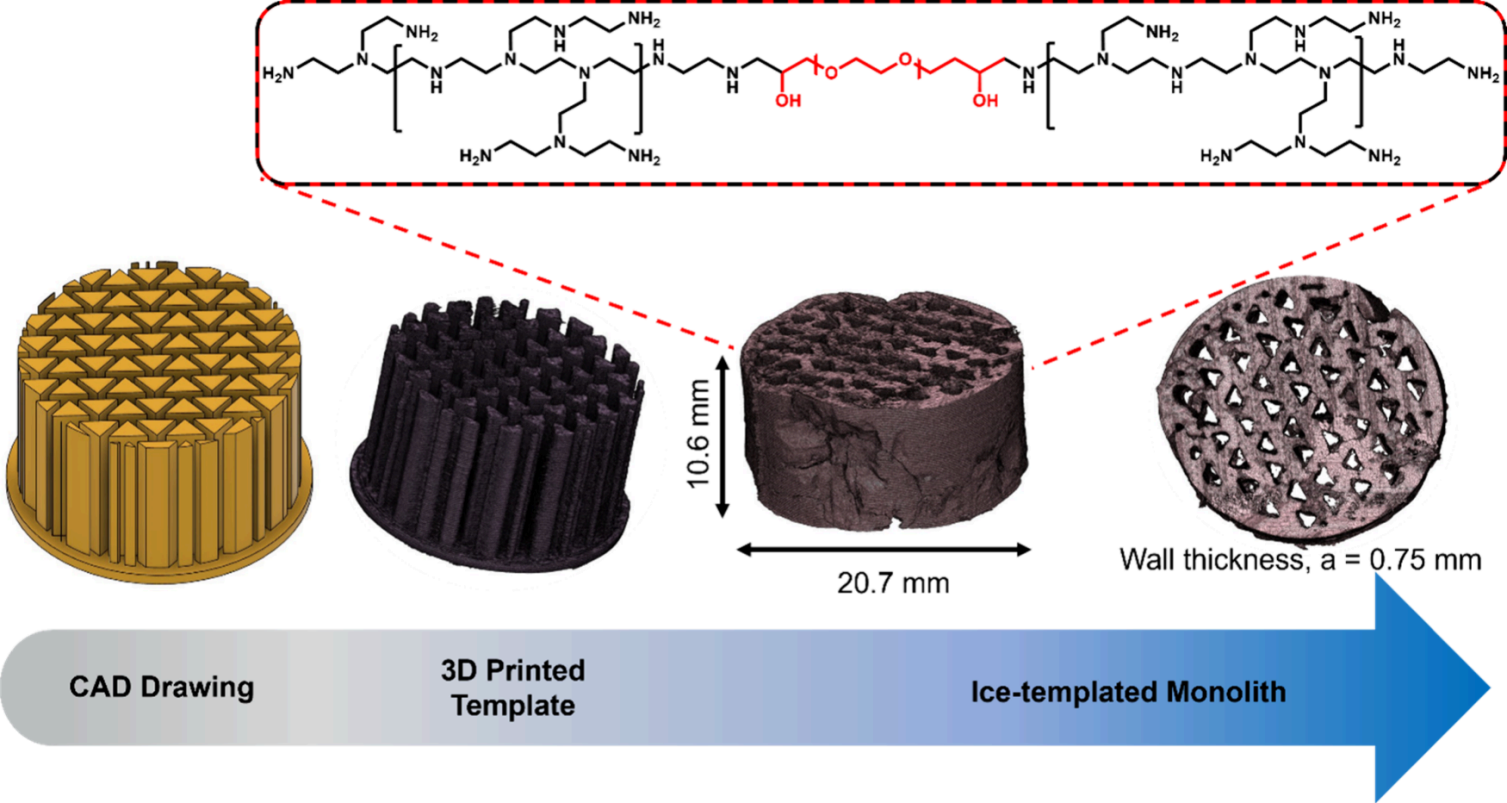
Representative process flow of PEI_196_2X showing the designed and 3D-printed template and the ice-templated PEI monolith (side and top views). The image of the 3D-printed template and the ice-templated monolith are from micro-CT. The inset shows the structure of cross-linked PEI.

The 3D structure of the monoliths was obtained by stitching together images of 350 layers, as obtained from micro-CT. The same was performed for the 3D-printed templates to evaluate the efficiency and resolution of the prints qualitatively. A stitched 3D model of PEI_196_2X and its template, along with an inset consisting of the structure of cross-linked PEI, is shown in Figure 3. A comparison of the initial CAD drawing and stitched 3D model of the printed template showed reasonable accuracy. The stitched 3D model of the cross-linked monolith was used to determine the actual monolith dimensions and shrinkage, depicted in Table 1. Despite the different channel wall thicknesses, the three monoliths exhibit similar overall dimensions and degree of shrinkage (about 52%) upon drying. Irregularities on the sides of the monoliths resulted from the monolith’s adhesion to the PLA template, which may be mitigated by further optimizing the printing parameters to obtain a smoother PLA template surface.

**Table 1:** Monolith Theoretical and Actual Dimensions and Volumetric Shrinkage

	theoretical from template		actual				
sample name	wall thickness (*a*_th_, mm)	channel density (*c*_th_, CPSI)	wall thickness (*a*, mm)^a^	monolith diameter (*d*, mm)^a^	monolith height (*H*, mm)^a^	volumetric shrinkage (Δ*V*, %)^a^	channel density (*c*, CPSI)
PEI_196_1X	0.59	79	0.41 ± 0.03	20.40 ± 0.17	10.62 ± 0.24	53.7 ± 1.3	132
PEI_196_2X	0.88	60	0.75 ± 0.10	20.74 ± 0.32	10.71 ± 0.13	51.9 ± 1.6	97
PEI_196_3X	1.17	48	0.97 ± 0.01	20.87 ± 0.35	10.70 ± 0.16	51.5 ± 1.4	76

a The actual dimensions are measured from SEM and Micro-CT images. The theoretical height and diameter are 13.7 and 26.4 mm, respectively.

**Figure 4:**
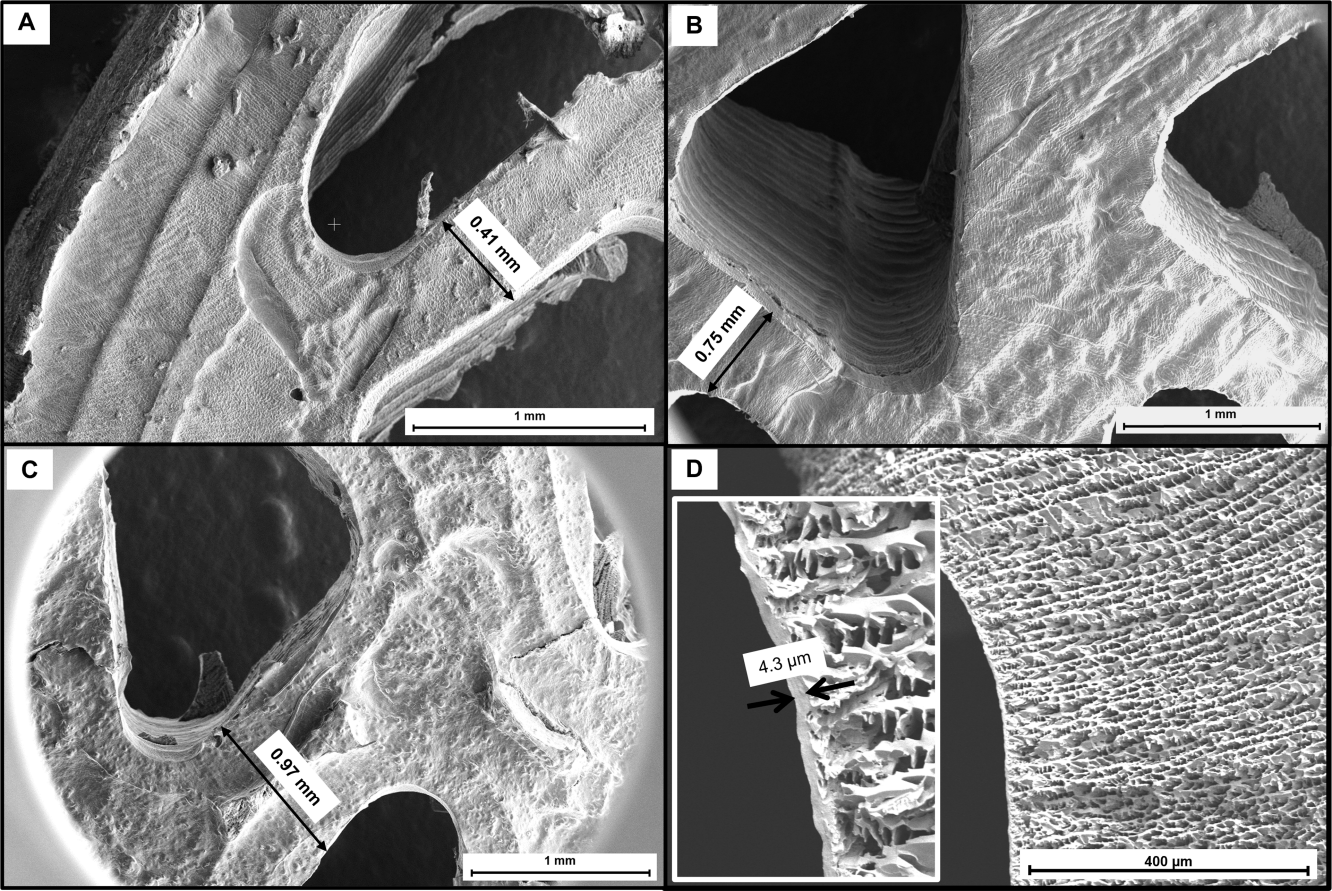
SEM images of the top layer of (a) PEI_196_1X, (b) PEI_196_2X, and (c) PEI_196_3X and (d) porous PEI_196_3X monolith sectioned from the bulk, with an inset showing the skin layer.

The morphology of the monolith surface was observed in SEM. The monolithic channel wall from the top layer of PEI_196_1X, PEI_196_2X, and PEI_196_3X and that of PEI_196_3X from an intermediate layer in the bulk are shown in parts a–d of Figure 4, respectively. The monolith channel wall thickness measured from the SEM using *ImageJ* software correlates with that measured from the micro-CT scans. From Figures 4a–c and S5, a semiporous “skin layer” is seen on the surface of the monoliths, including along the channel walls. This layer is formed from the expulsion of components other than water, i.e., PEI and PEGDGE in the aqueous phase. These components form a liquid film between the growing ice crystals and the interface (PVA surface), resulting in the skin layer.^84^ Meanwhile, ice nucleation and growth occur in the bulk solution, resulting in a uniform lamellar structure, as shown in Figure 4d, with a modal pore length between 5 and 10 μm that is typically seen in an ice-templated scaffold, as developed in previous works.^57,58,62,63,85^

The surface areas and the pore volumes of the adsorbents could not be measured with conventional, cryogenic nitrogen physisorption due to the macroporous structure and flexibility of the monoliths. However, useful information could be gleaned from the microscopy images obtained. The “skin layer” thickness in PEI_196_3X monolith from the inset in Figure 4d is 4.3 μm. The surface area of the skin layer increases with increasing channel density because the area of contact between the PEI and PEGDGE in the aqueous phase and the channel wall increases. A similar skin layer was found in polymer foams synthesized by Blaker et al. using temperature-induced phase separation, and this dense skin layer covering the porous substructure was found to increase the mechanical properties of the polymer foam.^86^ However, it is to be noted that such a skin layer can potentially result in the diffusional limitation of CO_2_ in the sorbent, as discussed in the following sections. The PEI–PEGDGE liquid film at the reactant mixture–PVA interface and the resulting dense skin layer can potentially be avoided by making design considerations to improve the rate of heat removal in the template, allowing for more rapid cooling and ice nucleation near the interface before the formation of a liquid film from expelled PEI and PEGDGE.

### CO_2_ Uptake: Effect of the Flow Rate

**Figure 5:**
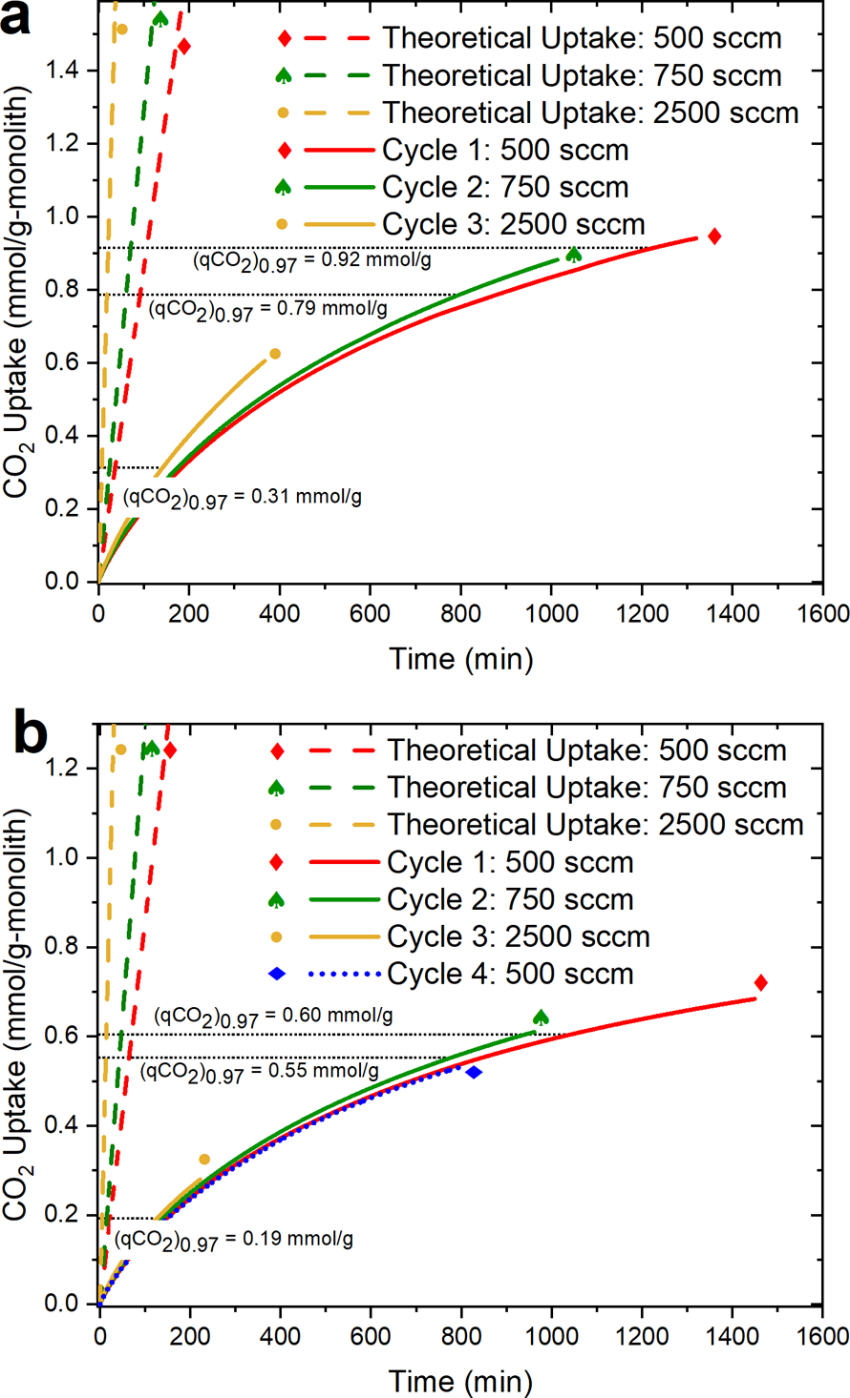
Integrated CO_2_ uptake in mmol/g of monolith of (a) PEI_196_1X and (b) PEI_196_3X at varying flow rates, represented by solid and dotted curves. Adsorption at 25 ± 0.2 °C from a feed containing 400 ppm of CO_2_/N_2_ and desorption at 100 °C using 250 sccm N_2_. Dashed lines represent theoretical CO_2_ uptake in the absence of kinetic limitation. (*q*_CO_2__)_0.97_ represents CO_2_ uptake of the monolith when the outlet CO_2_ concentration is 97% of the CO_2_ concentration in the feed. A custom-built dynamic breakthrough setup shown in Figure S3 was used.

Studying the effect of feed flow rate on the performance of the sorbents helps evaluate the external mass transfer. Dynamic breakthrough experiments were conducted using self-supported PEI monoliths at varying flow rates. The corresponding superficial velocity ranged from 2.5 to 12.5 cm/s. The breakthrough curves for PEI_196_1X and PEI_196_3X as a function of the product of time and air velocity are shown in Figures S6 and S7, respectively. The sorption rate or mass transfer from the gas phase to the sorption sites inside the monoliths greatly influences the shape of the breakthrough curves. Unlike a typical S-shaped breakthrough curve, the monoliths show an immediate breakthrough followed by a gradual increase in outlet CO_2_ concentration until equilibrium is achieved. This behavior indicates slow sorption kinetics such that a fraction of the CO_2_ molecules travel to the adsorber outlet before they can enter the pores of the sorbent. The slope of the breakthrough curve is indicative of the mass-transfer rates. The integrated CO_2_ uptake, *q*_CO_2__, is given by eq 1 and the same for PEI_196_1X and PEI_196_3X is shown in parts a and b of Figure 5, respectively. *F* is the feed flow rate, *y*_*i*_ indicates the molar fraction of CO_2_ in an empty bed or a bed containing the sample, and *mass* refers to the total dry mass of the monolith. The boundaries of integration *t*_0_ and *t*_f_ represent the beginning of the feed flowing into the module and the end of the experiment, respectively.
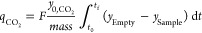
1

The red, green, and yellow solid curves in Figure 5a,b represent the integrated CO_2_ uptake from a feed gas with superficial velocities of 2.5 cm/s (500 sccm), 3.7 cm/s (750 sccm), and 12.4 cm/s (2500 sccm), respectively. The CO_2_ uptake shows some sensitivity toward the feed flow rate, with the uptake rate increasing with an increase in the flow rate. This behavior shows that there exists some external boundary layer resistance to the diffusion of CO_2_ within the monolith channels, and a further increase in the feed flow rate can improve the CO_2_ uptake rate. However, it should also be noted that such an increase in the feed flow rate will translate into a higher operating cost associated with the blowers.

The red, green, and yellow dashed lines are the theoretical CO_2_ uptakes (*q*_CO_2__)_th_ at 500, 750, and 2500 sccm, respectively. The theoretical CO_2_ uptake is given by eq 2, where *t*, *F*, and *C*_o_ are the adsorption time, feed gas flow rate, and concentration of CO_2_ in the feed gas, respectively.
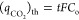
2

The deviation of the solid curves from the corresponding dashed lines is due to the presence of reaction kinetics and mass-transfer limitations. This deviation is common in sorbents because the theoretical uptake is for an ideal case that assumes there is no time spent by the CO_2_ molecules in reaching the amine sites, and that all the CO_2_ molecules from the feed gas entering the sorbent are adsorbed instantaneously. The theoretical uptake also assumes that when CO_2_ molecules reach the amine sites, they react with the amine groups instantaneously.^87,88^

(*q*_CO_2__)_0.97_, highlighted by the black dotted lines in the figure, represents the integrated CO_2_ uptakes until *C*/*C*_0_ reaches 0.97. The relationship between (*q*_CO_2__)_0.97_ and the feed flow rate for the monoliths is also shown in Figure S8. A lower (*q*_CO_2__)_0.97_ results from a longer tail in the breakthrough curve, while a higher (*q*_CO_2__)_0.97_ results from a shorter tail in the breakthrough curve. The (*q*_CO_2__)_0.97_ and the tail length become lower and longer, respectively, in the monoliths with an increase in the feed flow rate from 500 to 2500 sccm, indicating the existence of internal mass-transfer limitations. Meanwhile, the CO_2_ uptake curve of the first cycle (500 sccm) and that of the fourth cycle (also 500 sccm) for PEI_196_3X overlay on each other in Figure 5b, demonstrating the cyclic stability of the sorbent over four cycles.

### CO_2_ Uptake: Effect of Channel Density

**Figure 6:**
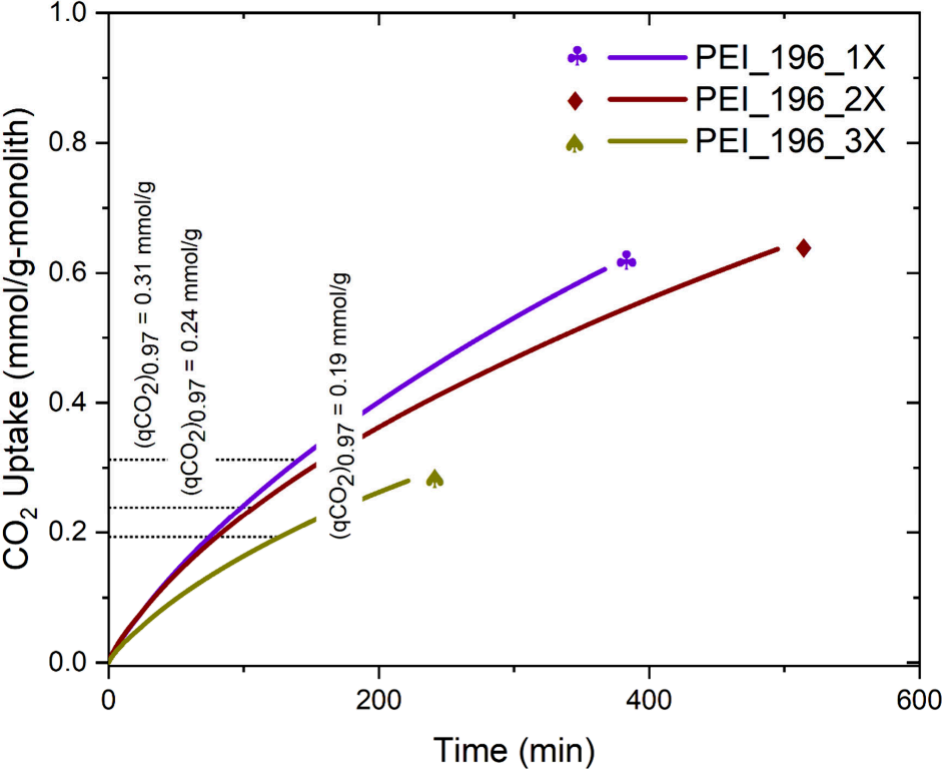
Integrated CO_2_ uptake in mmol/g of monolith of PEI_196_1X, PEI_196_2X, and PEI_196_3X at a constant flow rate of 2500 sccm. Adsorption at 25 ± 0.2 °C from a feed containing 400 ppm of CO_2_/N_2_ and desorption at 100 °C using 250 sccm N_2_. (*q*_CO_2__)_0.97_ represents the CO_2_ uptake the monolith when the outlet CO_2_ concentration is 97% of CO_2_ concentration in the feed. A custom-built dynamic breakthrough setup shown in Figure S3 was used.

The macroscopic and microscopic structure of the sorbent dictates the mass-transfer rate in the sorbent. The microscopic structure, including the pore size and pore wall thickness, have been studied in prior works and were found to be affected by the synthesis conditions such as the temperature and direction of freezing, freezing rate, degree of cross-linking, and the presence of additives.^52−54,89,90^ This work focuses on the macroscopic structure of the sorbent, which is defined by the channel density. The channel density influences the effective surface area of the monolith that is in contact with the feed stream and, in turn, affects the CO_2_ uptake rate. Studying the effect of channel density and wall thickness on the sorbents’ performance helps evaluate the internal mass transfer in the material. Three monoliths of different channel densities were studied for their CO_2_ capture performance, and the results are shown in Figure 6. The increase in the surface area with an increase in the channel density from monolith PEI_196_3X to PEI_196_1X resulted in an increase in the CO_2_ uptake rate. Varying the channel wall thickness affected the total number of channels, resulting in a change in the channel density. The CO_2_ uptake rate was more sensitive to changes in the channel density than to the flow rate, suggesting that internal mass transfer is more limiting than the external mass transfer in these monoliths under the study conditions. Moreover, based on the (*q*_CO_2__)_0.97_ (Figure S8), the tail in the breakthrough curve gets sharper with thinner channel walls due to its counter effect on internal mass-transfer limitations.

From Figure 5, the CO_2_ uptake rate in PEI_196_1X is more sensitive to an increase in the flow rate than it is in PEI_196_3X. PEI_196_1X being a monolith with more external surface area per unit volume (74% more than that of PEI_196_3X) can accommodate more CO_2_ molecules on the surface, while the external surface of PEI_196_3X may become saturated with CO_2_ molecules more easily at high feed flow rates. The surface saturation is further enhanced by the lower CO_2_ penetration rate into the thicker walls of PEI_196_3X because of internal mass-transfer limitations. Therefore, PEI_196_1X shows potential for improvement in the CO_2_ uptake rate with an increase in the feed flow rate. The slower penetration of CO_2_ also results in an unused “dead zone” in the monolith. The volume of this dead zone is smaller in PEI_196_1X than in PEI_196_3X due to a higher external surface area and thinner channel wall in the former, enabling a better CO_2_ uptake rate in PEI_196_1X.

### Effect of Oxidation

**Figure 7:**
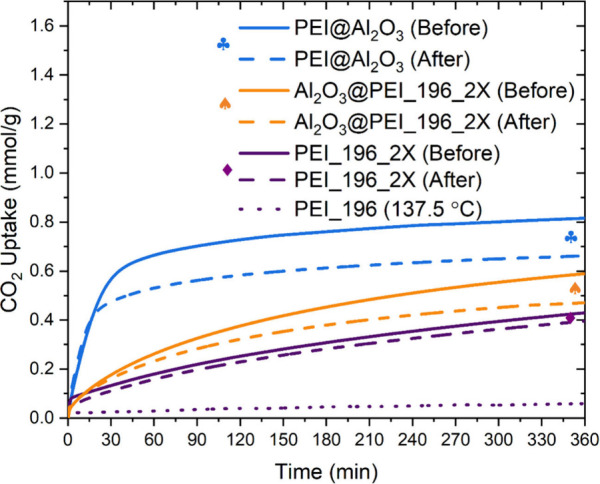
Thermogravimetric CO_2_ uptake of 35 wt % PEI impregnated on Al_2_O_3_ (PEI@Al_2_O_3_), self-supported PEI monolith with 6.5 wt % Al_2_O_3_ as an additive (Al_2_O_3_@PEI_196_2X), and self-supported PEI monolith (PEI_196_2X) before (solid curves) and after exposure to 90 sccm 21% O_2_/N_2_ at 110 °C (dashed curves) or 137.5 °C (dotted curve) for 3 h. Adsorption at 25 °C from a feed flowing at 90 sccm.

Oxidative degradation of amines is one of the pressing challenges to their practical application in DAC.^91−96^ While there are continued efforts toward understanding the mechanism of degradation,^93,97−99^ there is a need for simultaneous development of oxidation-resistant materials based on the gained knowledge. It is also essential to evaluate the performance of existing sorbents under harsh oxidative conditions. To this end, the effect of oxidation on the performance of the self-supported b-PEI sorbent has been evaluated. The loss in CO_2_ uptake after exposure to an oxygen-containing environment is regarded as a degradation metric for this study. The performance of the monoliths in this work has been benchmarked by comparing against a well-known system, b-PEI impregnated on an inorganic Al_2_O_3_ support (PEI@Al_2_O_3_), shown in Figure 7. The PEI impregnated on Al_2_O_3_ sorbent initially had the highest CO_2_ uptake, of 0.82 mmol/g of sorbent, after a 6 h exposure to CO_2_ at 25 °C. In contrast, the self-supported b-PEI monolith (PEI_196_2X) had the lowest uptake among the sorbents studied, due to the smaller number of primary amines and more secondary and tertiary amines in the b-PEI monolith than the impregnated PEI sorbents. This change in amine distribution is due to the conversion of some primary amines to secondary and further into tertiary amines during the cross-linking of PEI to synthesize the self-supported PEI sorbents.^52−54,63^ The amine distribution for a small scaffold has been estimated in a prior work.^53^

The cross-linked PEI monoliths have a comparatively dense cross-linked network, offering more resistance to the CO_2_ molecules reaching the free amine sites. The CO_2_ uptake rate and the 6-h CO_2_ adsorption capacity increase with the addition of small amounts of γ-Al_2_O_3_ to the ice-templating process (Al_2_O_3_@PEI_196_2X). This enhancement in the performance of the monolith follows the same trend as seen in the PEI scaffolds in our previous work, possibly due to the reduced pore size and pore wall thickness.^54^

The loss in CO_2_ uptake after exposure to harsh oxidative conditions is higher in sorbents with higher Al_2_O_3_ content and vice versa. The better retention of CO_2_ uptake by the PEI_196_2X monolith than by the sorbents with Al_2_O_3_ could potentially be attributed to several factors, including (i) the formation of hydrogen bonds between the hydroxyl groups and amine moieties in the cross-linked PEI monolith, reducing the accessibility of oxygen to amines,^100,101^ (ii) the possibility of metal contaminants in the commercial Al_2_O_3_ used, which could catalyze the oxidation reaction, and (iii) the lower diffusivity of O_2_ in the cross-linked PEI network than in the PEI impregnated sorbent. The PEI-impregnated sorbent, PEI@Al_2_O_3_, has the highest CO_2_ uptake kinetics and possibly oxygen diffusivity, showing the highest oxygen-induced degradation among the sorbents. Similarly, Al_2_O_3_@PEI_196_2X likely has an intermediate oxygen diffusion resistance, resulting in an intermediate degradation level. When the same sorbents were further exposed to highly harsh oxidative conditions at 137.5 °C, they underwent complete degradation and could adsorb only a negligible amount of CO_2_ after the exposure. It is to be noted that such a temperature is beyond the range of regular operation in the CO_2_ capture process and was used to accelerate degradation.

### CO_2_ Adsorption from Ambient Air

Most DAC studies have been carried out using simulated air of 400 ppm of CO_2_ balanced by an inert gas such as He or N_2_, with or without humidity.^17,20,61,102−105^ In some cases, the relevant oxygen concentration is added to the simulated air mixture to study oxidation effects.^91,92,98,100,106,107^ Ambient air contains several other contaminants that are difficult to mimic in lab-scale studies, including aerosols, dust, pollen, ozone, and more.^108^ However, it is essential to understand the performance of the sorbents under such realistic conditions.

**Figure 8:**
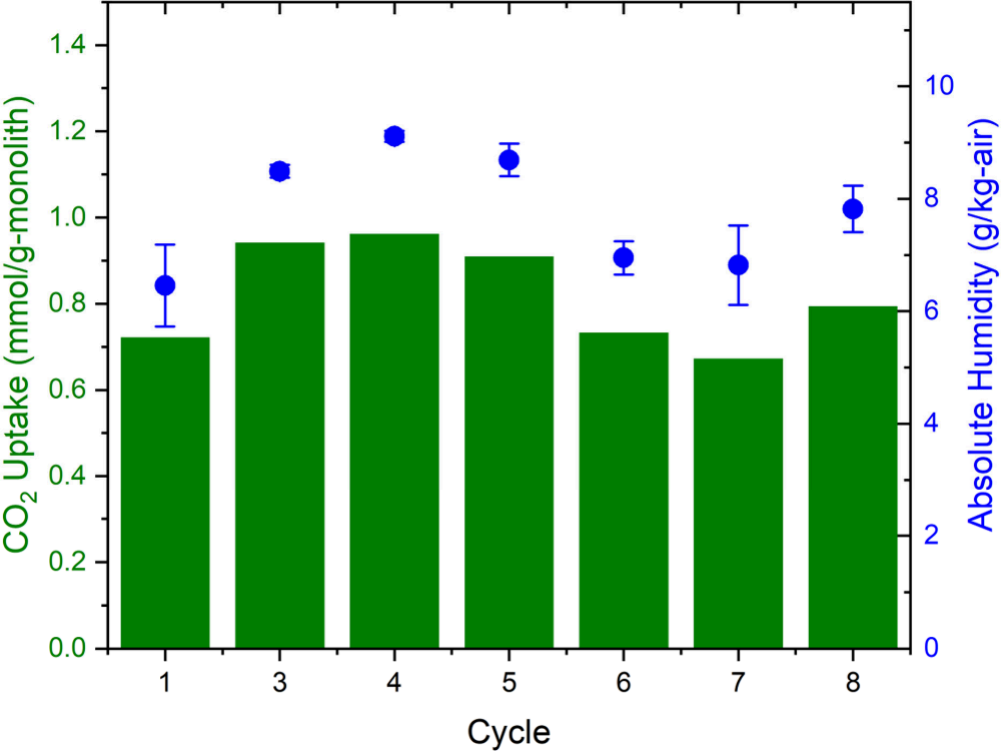
CO_2_ uptake in mmol/g of PEI_196_2X monolith across eight cycles from real ambient air containing varying humidity levels after presaturation with saturated KCl solution (∼85% RH). Adsorption was for 12 h at 24.8 ± 0.5 °C from 500 sccm ambient air containing 448 ± 7 ppm of CO_2_, followed by desorption under 500 sccm N_2_ at 100 °C until no CO_2_ or water was detected downstream. CO_2_ uptake and absolute humidity (g/kg of air) are represented by green bars and blue circles, respectively.

Monolith PEI_196_2X was subjected to eight adsorption–desorption cycles with an adsorption flow rate of 500 sccm using ambient room air. A water presaturation step was included between them to deconvolute the competitive effects of water and CO_2_ and mimic sorbent regeneration using steam. The 12 h CO_2_ uptake for cycles 1–8 is presented in Figure 8, along with the corresponding average absolute humidity across the duration of adsorption. Cycle 2 is not presented, as it was performed at a different flow rate. Despite the fluctuations in CO_2_ uptake across the adsorption cycles, there is no noticeable declining trend. The CO_2_ uptake fluctuations correlate with the feed gas’s humidity fluctuation, potentially due to the enhanced theoretical amine efficiency and improved diffusion of CO_2_ molecules through the cross-linked PEI network in the presence of increased humidity.^109,110^ A maximum CO_2_ uptake of 0.96 mmol/g of monolith was achieved, corresponding to an absolute humidity of 9.11 g of H_2_O/kg of air (i.e., 45.5% RH at 25 °C). This CO_2_ uptake from ambient air was higher than that from a 400 ppm of CO_2_ cylinder under dry conditions. This increased uptake could be attributed to the improved diffusion of CO_2_ in the presence of humidity.^52,111^ The outlet CO_2_ concentration reached 0.95 times the inlet CO_2_ concentration after 12 h. Practical, DAC units require much shorter adsorption times for reasonable productivity. However, the velocities used in this experiment are significantly lower (by a factor of 50) than those used in practical DAC systems; Higher CO_2_ delivery rates are expected to reduce the adsorption step time dramatically. Based on the effect of feed flow rate and channel density on the CO_2_ uptake rate discussed earlier, a higher channel density and thinner channel walls should enable similar uptake in shorter adsorption times at constant velocity. Eliminating or reducing the thickness of the skin layer on the monolith surface can further improve CO_2_ diffusion and reduce the required adsorption time. It is noted that both of these could affect the mechanical stability of the sorbent.

### Pressure Drop

**Figure 9:**
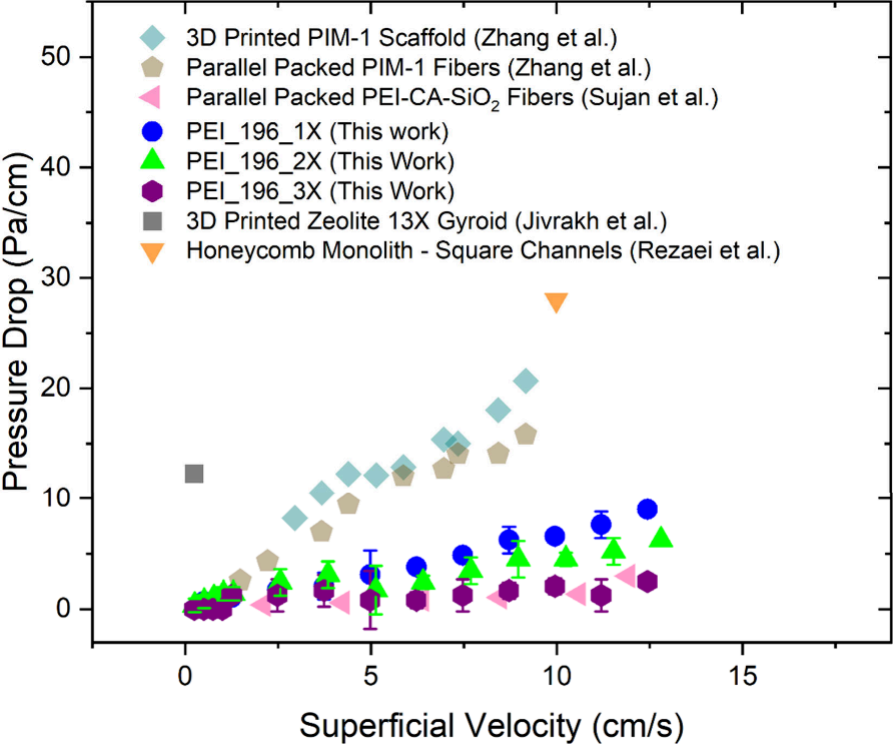
Pressure drop across PEI_196_1X (blue circle, 132 CPSI), PEI_196_2X (green triangle, 97 CPSI), and PEI_196_3X (maroon hexagon, 76 CPSI) modules compared to that of a 3D-printed PIM-1 scaffold (light blue diamond, by Zhang et al. Figure adapted with permission from ref (115). Copyright 2009 John Wiley and Sons), parallel packed PIM-1 fibers (beige pentagon, by Zhang et al. Figure adapted with permission from ref (115). Copyright 2018 John Wiley and Sons), parallel packed PEI–cellulose acetate–SiO_2_ fibers (pink left-pointing triangle, by Sujan et al. Figure adapted with permission from ref (104). Copyright 2019 American Chemical Society), 3D-printed zeolite 13X gyroid (gray square, by Jivrakh et al. Figure adapted with permission from ref (67). Copyright 2024 Elsevier), and honeycomb monolith with square channels equivalent to commercial ceramic monoliths (200 CPSI, orange inverted triangle, by Rezaei et al. Figure adapted with permission from ref (114). Copyright 2009 Elsevier) at varying superficial velocity.

The ultradilute concentration of CO_2_ in ambient air means that a larger volume of air would have to be processed to capture the same amount of CO_2_ as from a comparatively more concentrated source such as flue gas. Hence, for the practical application of DAC, minimizing the pressure drop to reduce energy consumption in moving the air and the associated operating cost is essential. The configuration of the sorbent used can significantly impact the pressure drop associated with the airflow by affecting the tortuosity and viscous drag force.^45,112^Figure 9 shows the pressure drop of the three monoliths studied as a function of superficial velocity. The highest achievable superficial velocity in the setup was 12.8 cm/s, corresponding to a flow rate of 2500 sccm. PEI_196_3X showed the smallest pressure drop among the sorbents studied, while PEI_196_1X experienced the highest pressure drop. This observation is per the trend followed by the channel density and pressure drop in literature,^45,112−114^ with PEI_196_3X having the lowest channel density and PEI_196_1X having the highest channel density, among the monoliths developed. Overall, the pressure drop across the self-supported b-PEI monoliths was in the same range as and even considerably lower than some of the fibers, 3D-printed contractors, and the honeycomb monolith with straight channels in the literature.^67,104,114−116^

### Mechanical Properties

The as-prepared monoliths were converted to a swollen state when immersed in water. The percent swelling based on the increase in the overall volume of the monolith was the same as the volume lost during the thawing and drying stages of monolith synthesis. Yoo et al. observed a similar swelling behavior when they immersed b-PEI scaffolds in water.^52^ This swelling behavior can be attributed to the water-induced dilation of the cross-linked polymer networks that otherwise exist in a collapsed and more dense state under dry conditions. This is also evidenced by the increased fragility of the monoliths in aqueous media. However, the same was not observed in the presaturation step of CO_2_ uptake experiments after equilibration with humidity of up to 85% RH at 25 °C, corresponding to 19.96 g of water/kg of air. The monoliths, from observation, showed elastic behavior under ambient conditions of temperature and humidity.

**Figure 10:**
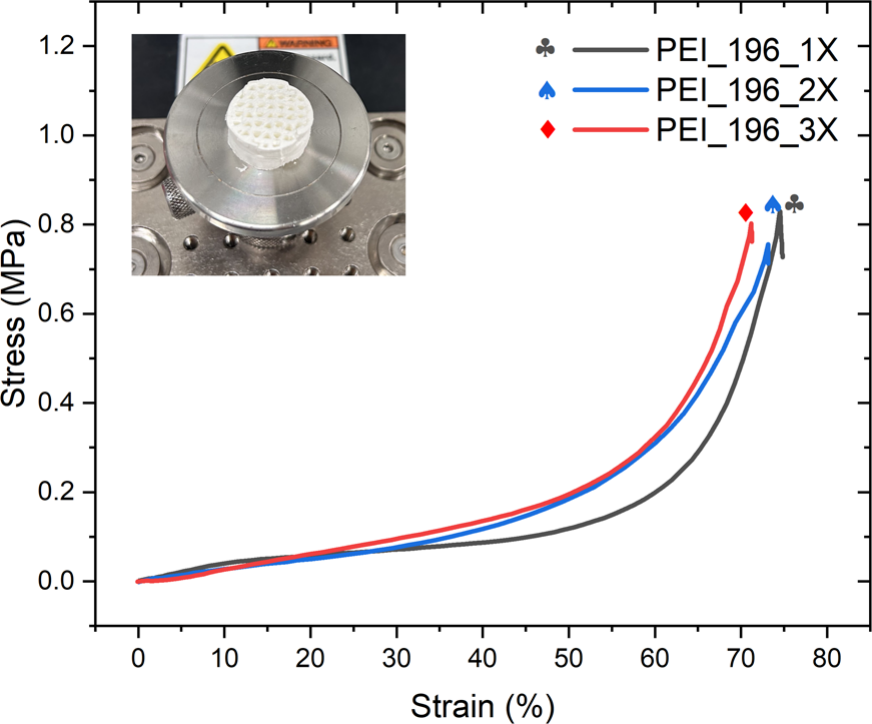
Stress–strain curves of PEI_1X, PEI_2X, and PEI_3X when a 250 N rated force gauge was lowered at 10 mm/min until fracture. The inset shows PEI_196_3X after the compression test.

To further understand the mechanical strength of the monoliths, PEI_1X, PEI_2X, and PEI_3X sorbents were subjected to compression by a mechanical tester. The stress vs strain curves are shown in Figure 10. The monoliths show a similar compressive strength of ∼0.8 MPa. Despite a few cuts in the monoliths, they completely recovered to their original size when the load was removed. PEI_196_3X undergoes fracture at a slightly lower strain (70%) than PEI_196_1X (74%) and PEI_2X (72.5%), due to its thicker channel wall restricting the deformation. The monoliths can withstand reasonable stress and undergo elastic deformation without being brittle. It is noted here that the degree of cross-linking and cross-linker chain length impact the elasticity of the monolith. Previous attempts at cross-linking PEI with epoxides of shorter chain lengths than PEGDGE have yielded brittle sorbents.^63^ A comparison of the compressive strength of self-supported PEI monoliths fabricated in this work, against that of some of the monoliths in literature for CO_2_ capture presented in Table S1 highlights the promising mechanical stability of self-supported PEI monoliths.

## Conclusions

Ice templating and 3D printing were used to develop self-supported amine monoliths for DAC. The channel density was varied, and dynamic breakthrough experiments were conducted in simulated dry air and ambient room air containing humidity. The monoliths have superior oxidation stability compared to an amine-impregnated alumina sorbent as well as good mechanical stability. They were also found to have a low pressure drop among the sorbents and fibers reported in the literature. The maximum CO_2_ uptake under ambient air containing 45% RH was 0.96 mmol/g of monolith. While this CO_2_ uptake is on par with and superior to some amine functionalized sorbents in the literature, the sorbents studied exhibit mass-transfer limitations, indicating the need for further design improvements.^102,105,117^ The positive effect of a higher flow rate shows the presence of meaningful external mass-transfer resistances. Increasing the feed flow rate will likely increase the CO_2_ uptake rate until the external resistance to mass transfer is minimized, followed by which, the impact of the flow rate would plateau. Moreover, the sensitivity of CO_2_ uptake toward channel density implies that the internal mass transfer is more limiting than external mass transfer under the conditions investigated. This impact showcases the need for further macroscopic and microscopic design development in the material for improved performance. Reducing the channel wall thickness and developing more complex geometries for the contactors such as triply periodic minimal surface can potentially improve the mass-transfer rate. Additionally, incorporating a small amount of additives in the ice templating can affect the morphology of the pores formed, and thereby can positively impact the mass transfer in self-supported PEI monoliths.^54,118^

Nevertheless, this work serves to demonstrate the potential of self-supported amine monoliths with well-defined structures and good sorbent stability for applications in CO_2_ capture from ultradilute sources. Also, it should be noted that currently, most reported DAC sorbents exist in powder form. A 35 wt % PEI-impregnated Al_2_O_3_ powdered sorbent has an amine loading of 6.3 mmol of N/g of sorbent.^54^ In comparison, the amine loading of such self-supported cross-linked PEI sorbents developed using ice templating is 8.4 mmol of N/g of monolith.^53^ The traditionally used powdered sorbents would have to be shaped into structures and pellets using binders and additives for practical application. The additional components reduce the effective amine loading of the structured contactors and may diminish the performance of the sorbent. However, in this work, the sorbent exists by itself, and all the measurements made can more accurately translate to actual application.^53^

One of the limitations of this work is an unrealistic regeneration process with inert gas. Steam stripping would more likely be employed in practice, and the water-induced swelling of the sorbent would have to be monitored and controlled. Additionally, the role of the skin layer should be investigated further, and efforts should be dedicated to improving the heat removal rate in ice templating, especially during attempts to lower the diffusion length by using thinner channel walls.

## Supporting Information Available

Printing parameters for PLA and PVA templates, schematic of the setup used for adsorption experiments and for determination of the pressure drop, SEM of the skin layer in PEI_196_3X, normalized breakthrough curves for PEI_196_1X and PEI_196_3X with varying air velocities, and (*q*_CO_2__)_0.97_ at different feed flow rates for monoliths PEI_196_1X, PEI_196_2X, and PEI_196_3X (PDF)
STEP file for the PLA casing and PVA inverse templates (ZIP)
