# Supplementary material for: Self-Supported Branched Poly(ethylenimine) Monoliths from Inverse Template 3D Printing for Direct Air Capture
Source: ACS Appl Mater Interfaces. 2025 Feb 11;17(7):10696–709. doi: 10.1021/acsami.4c20617 (PMC11843543; doi:10.1021/acsami.4c20617)
Supplement: Supplementary file 1 — am4c20617_si_001.pdf [file am4c20617_si_001.pdf]

Supporting Information for:

Self-Supported Branched Poly(ethyleneimine) Monoliths from Inverse Template 3D Printing for Direct Air Capture

Pavithra Narayanan, Seo-Yul Kim, Dema Alhazmi, Christopher W. Jones,* Ryan P. Lively,*

School of Chemical & Biomolecular Engineering, Georgia Institute of Technology,

Atlanta, Georgia, 30332, United States

Corresponding authors:

*Email: cjones@chbe.gatech.edu

*Email: ryan.lively@chbe.gatech.edu

List of Figures

S. No.	Title of Figure	Pg. No.
S1	Figure S1: Printing parameters for PLA outer shell with a sample pre-print image.	SI-4
S2	Figure S2: Printing parameters for PVA sacrificial template with a sample pre-print image.	SI-5
S3	A schematic of the dynamic breakthrough adsorption experiments used to study CO_2 uptake in self-supported PEI monoliths with insets showing module and monolith in module.	SI-7
S4	A schematic of the experimental setup to determine pressure drop.	SI-8
S5	SEM of the skin layer along the channel wall in PEI_196_3X	SI-9
S6	(a) Normalized breakthrough curves with varying superficial air velocities for PEI_196_1X with (b) showing the same at shorter time scale. CO_2 concentration = 400 ppm, T = 25 ± 0.2 °C	SI-10
S7	(a) Normalized breakthrough curves with varying superficial air velocities for PEI_196_3X with (b) showing the same at shorter time scale. CO_2 concentration = 400 ppm, T = 25 ± 0.2 °C	SI-11
S8	$(qCO_2)_{0.97}$ at different feed flow rates for monoliths PEI_196_1X (●), PEI_196_2X (▲) and PEI_196_3X (♦). $(qCO_2)_{0.97}$ is the integrated CO_2 uptake corresponding to a C/C_0 of 0.97 i.e. CO_2 uptake when the outlet CO_2 concentration is 97% of the CO_2 concentration in the feed. Adsorption at 25 ± 0.2 °C from a feed containing 400 ppm CO_2/N_2 and desorption at 100 °C using 250 sccm N_2.	SI-12

List of Tables

S. No.	Table Caption	Pg. No.
S1	Table S1: Comparison of mechanical property of self-supported PEI monolith with other sorbents in literature	SI-13

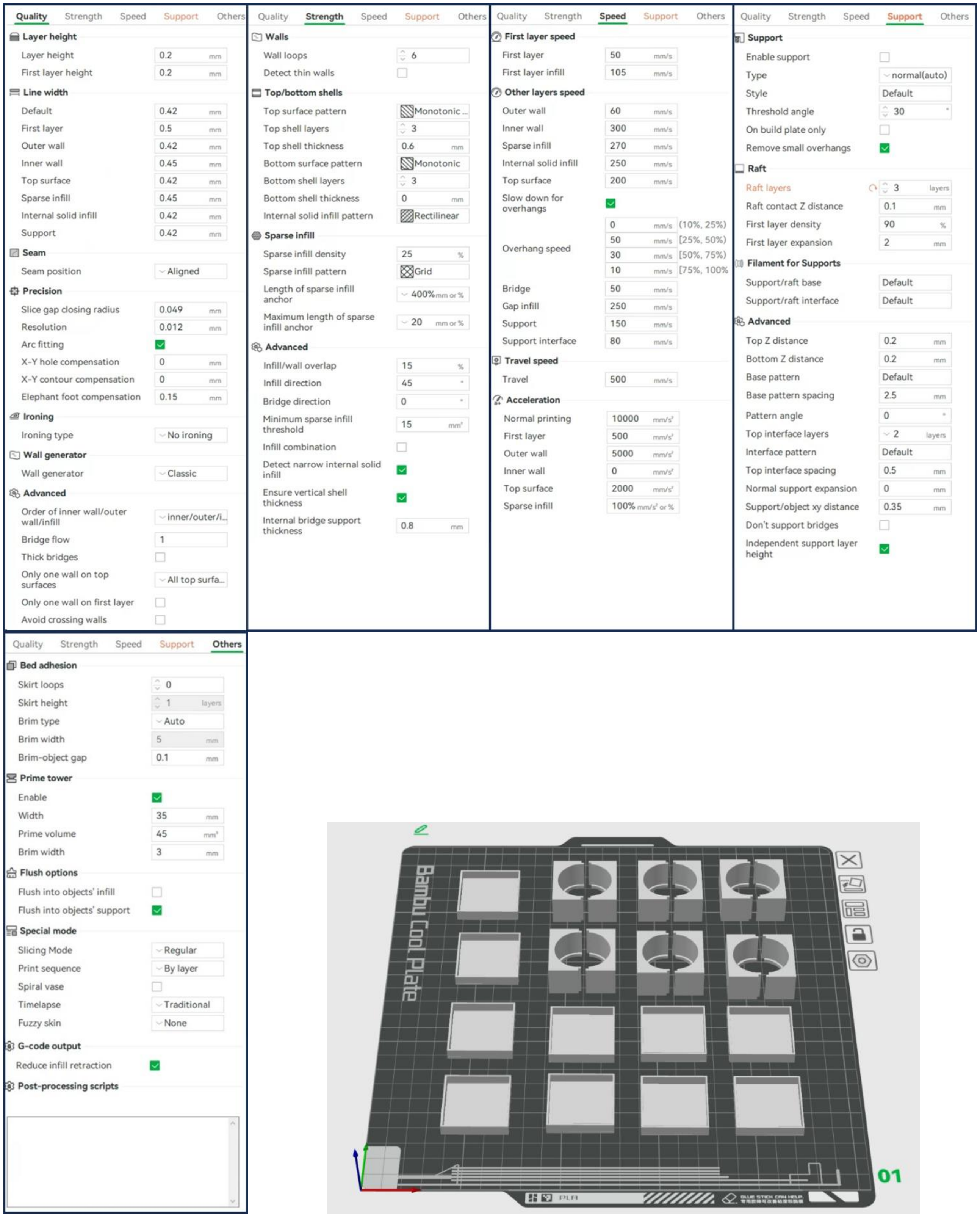

Figure S1: Printing parameters for PLA outer shell with a sample pre-print image.

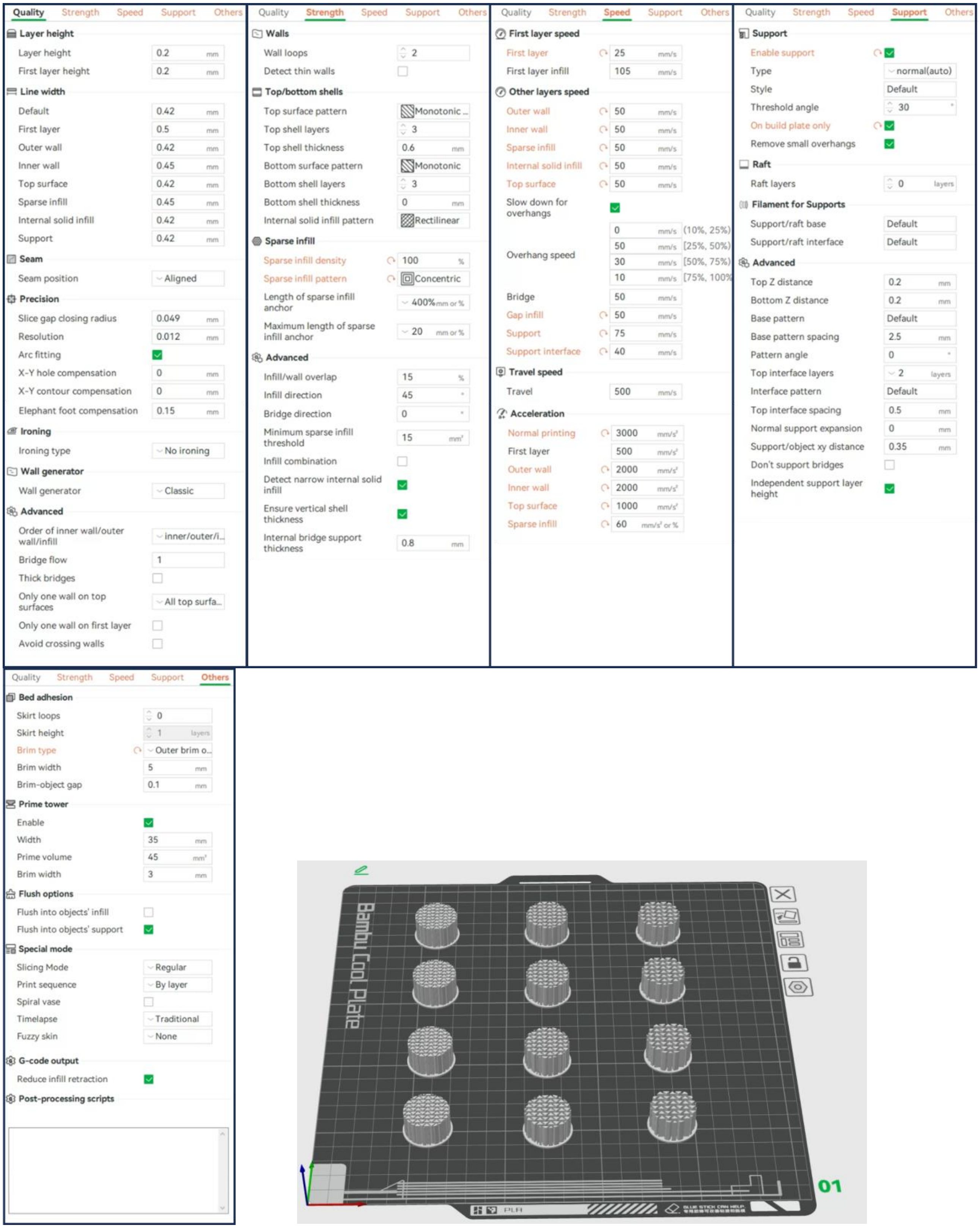

Figure S2: Printing parameters for PVA sacrificial template with a sample pre-print image.

Section S2: Dynamic Breakthrough Setup

The experimental design described below was used for the dynamic CO_2 adsorption breakthrough studies from dry 400 ppm CO_2/N_2 (dry) and indoor air.

CO_2 adsorption experiments were performed in a custom-built fixed bed. A schematic of the setup is shown in **Figure S3**. The setup consists of N_2 and 400 ppm CO_2/N_2 cylinders for CO_2 uptake experiments from dry streams. The experiments with adsorption from indoor air used a Dayton blower as the feed gas source. An Inkbird CO_2 sensor was used to record the ambient temperature, concentration of CO_2 in the room, and relative humidity every minute during the adsorption process. All the gas flow rates were controlled using Alicat mass flow controllers (MFCs). Two thermocouples – one measuring the temperature on the outer surface of the module and controlling the heat tape and the other reading the temperature on the surface of the monolith inside the module were used for temperature control. LiCOR, an IR gas analyzer, measured the CO_2 and water concentration downstream. While ambient air was used for humid adsorption experiments, humid N_2 was required for the pre-saturation step. Relative humidity of ~85% RH was generated by passing dry N_2 gas through a bubbler containing a saturated potassium chloride (KCl) solution. A chiller operating with a 50:50 water and ethylene glycol mixture flowing in stainless-steel tubing wound around the module was used to aid in the cooling step after activation and desorption. An Agilent ADM flowmeter was connected to the inlet of the bed or outlet of the LiCOR (as needed) to measure each gas stream's flow rate accurately.

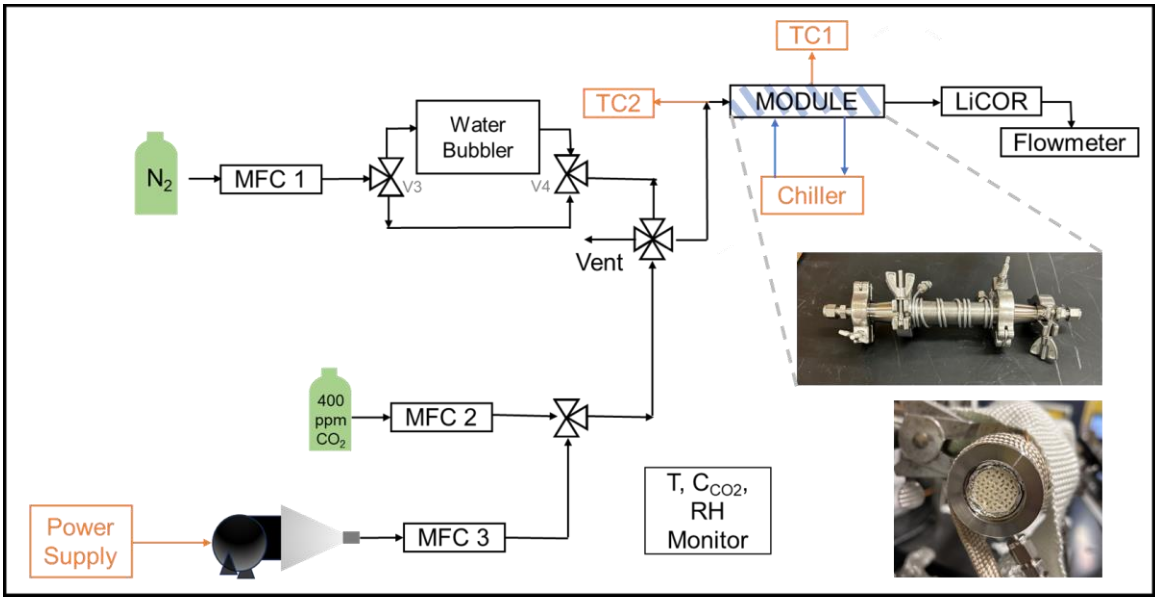

Figure S3: A schematic of the dynamic breakthrough adsorption experiments used to study CO_2 uptake in self-supported PEI monoliths with insets showing module and monolith in module.

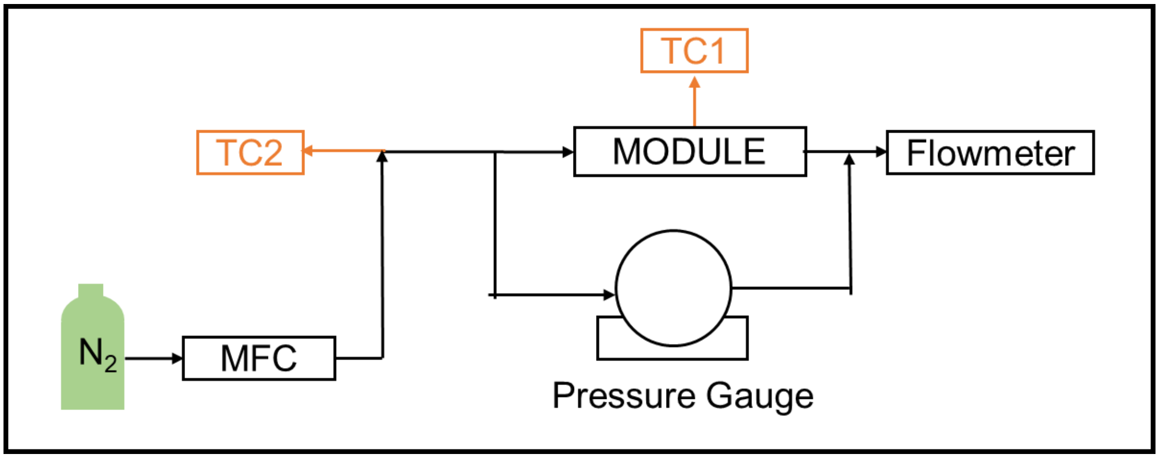

Figure S4: A schematic of the experimental setup to determine pressure drop.

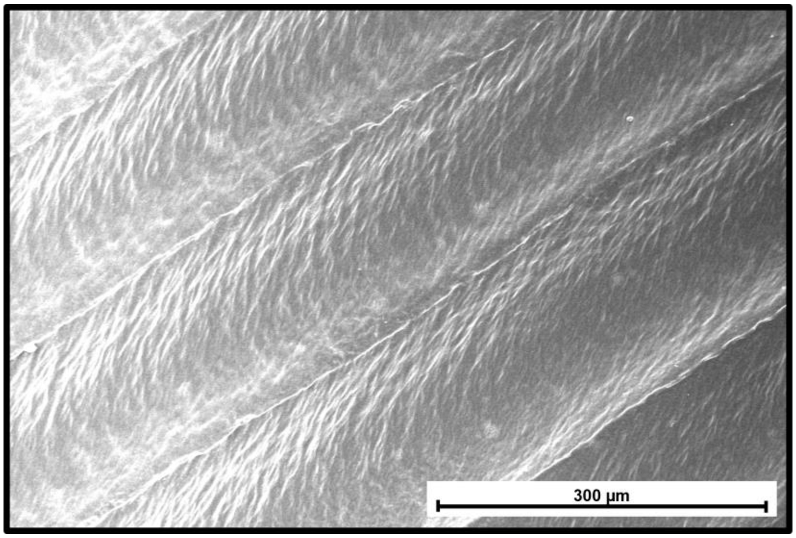

Figure S5: SEM of the skin layer along the channel wall in PEI_196_3X

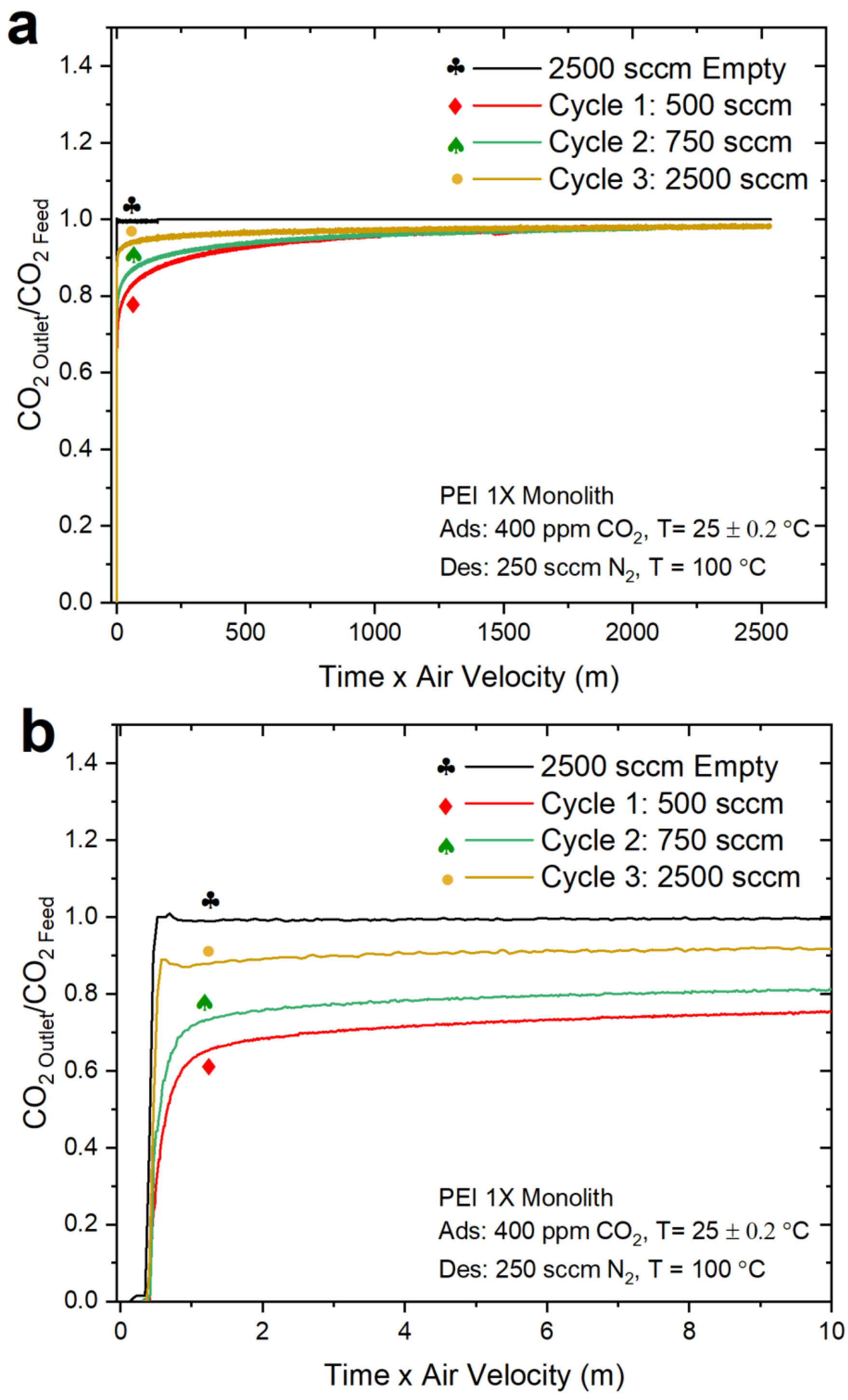

Figure S6: (a) Normalized breakthrough curves with varying superficial air velocities for PEI_196_1X with (b) showing the same at shorter time scale. CO_2 concentration = 400 ppm, T = 25 ± 0.2 °C

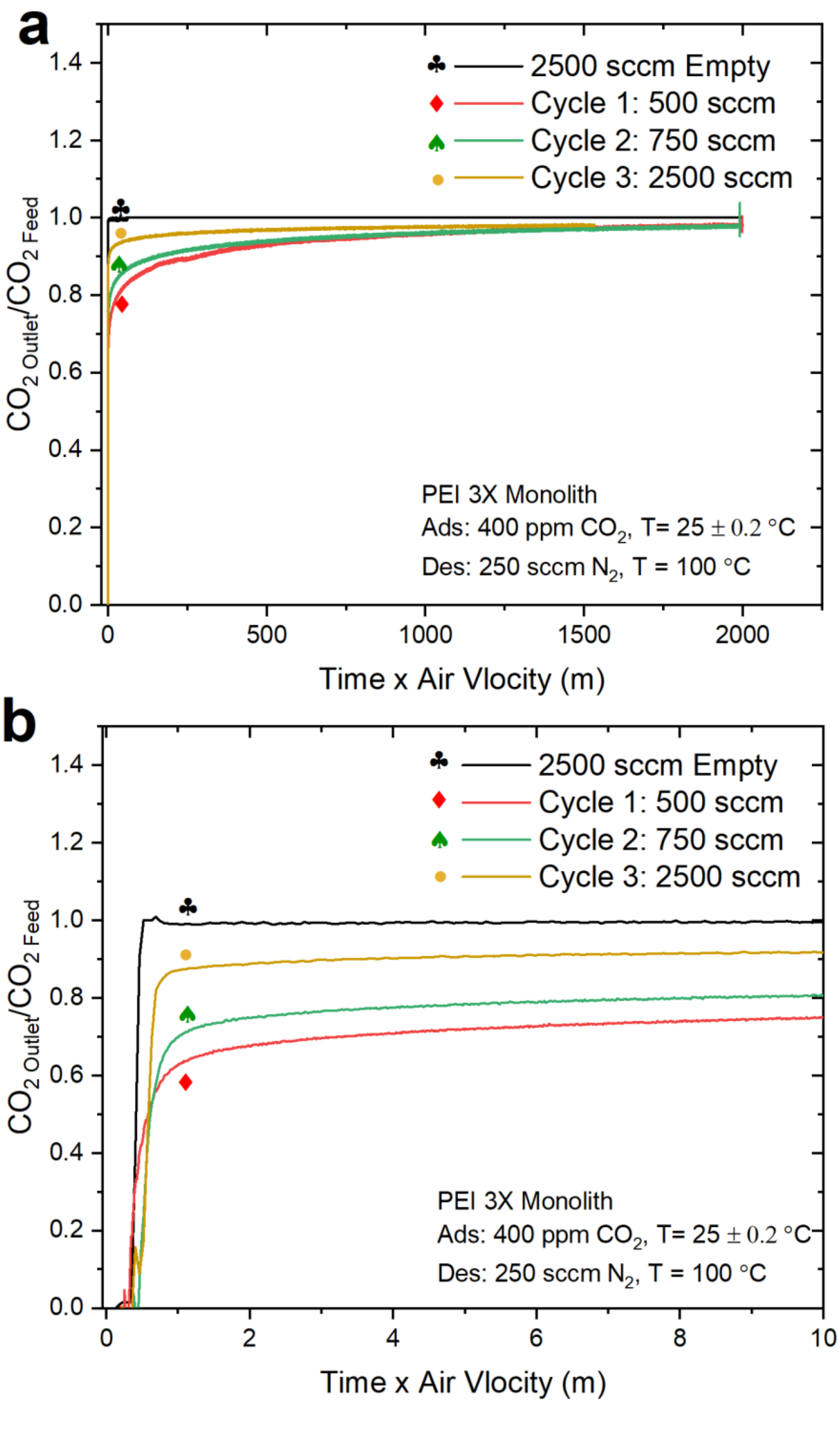

Figure S7: (a) Normalized breakthrough curves with varying superficial air velocities for PEI_196_3X with (b) showing the same at shorter time scale. CO_2 concentration = 400 ppm, T = 25 ± 0.2 °C

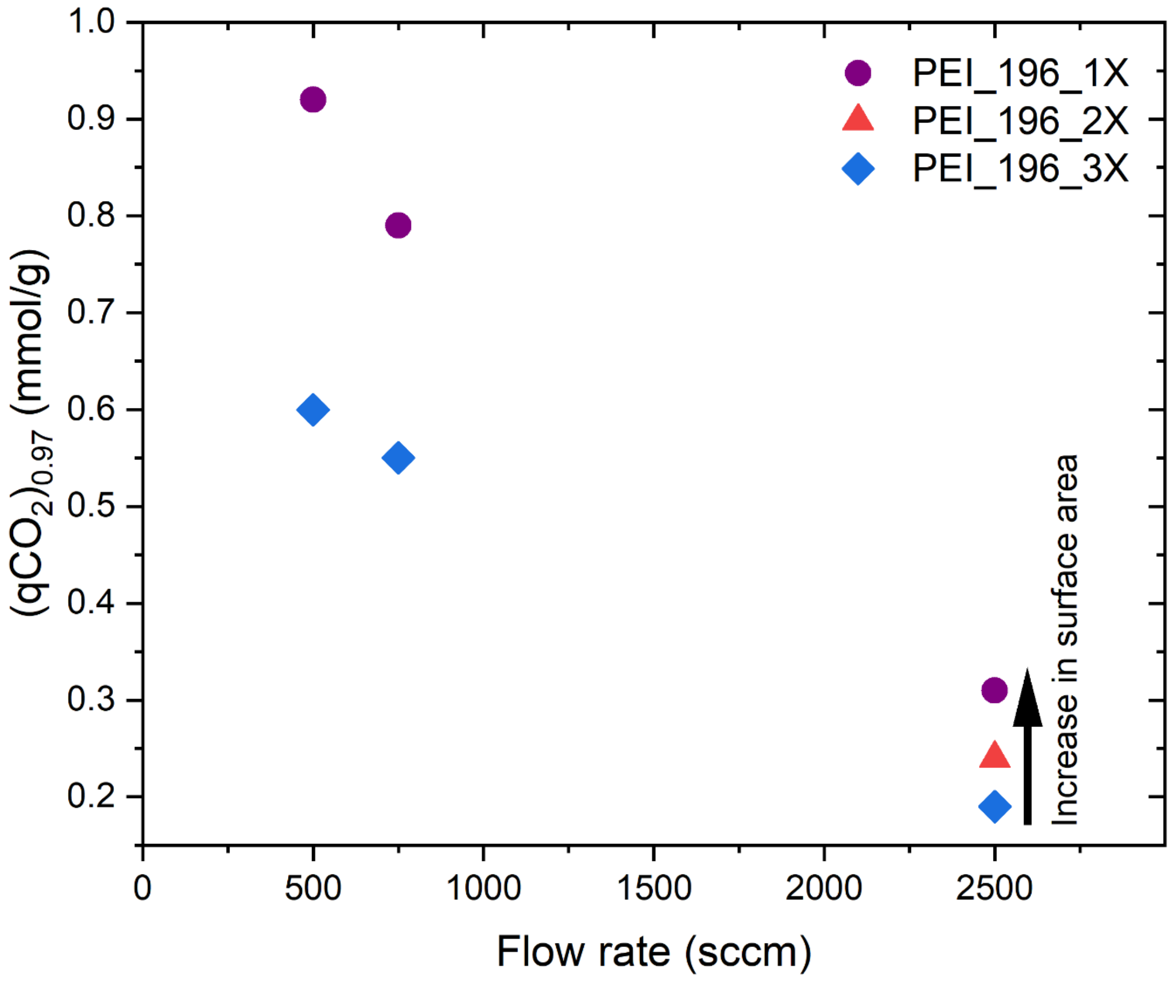

Figure S8: $(qCO_2)_{0.97}$ at different feed flow rates for monoliths PEI_196_1X (●), PEI_196_2X (▲) and PEI_196_3X (♦). $(qCO_2)_{0.97}$ is the integrated CO_2 uptake corresponding to a C/C_0 of 0.97 i.e. CO_2 uptake when the outlet CO_2 concentration is 97% of the CO_2 concentration in the feed. Adsorption at 25 ± 0.2 ˚C from a feed containing 400 ppm CO_2/N_2 and desorption at 100 ˚C using 250 sccm N_2.

Table S1: Comparison of mechanical property of self-supported PEI monolith with other sorbents in literature

Sorbent	Form Factor	Compression rate	Compressive Strength	Ref
Crosslinked PEI	Monolith with triangular channels	10 mm/min	0.8 MPa	This work
Hierarchical carbon cryogel	Cylindrical	0.10 /s*	0.17 MPa – 0.67 MPa	Li et al.[1]
Urea-modified carbon cryogel	Cylindrical	Unknown	0.62 MPa	Nazir et al.[2]
Zeolite 13X	Cylindrical with square channels	2.5 mm/min	0.30 MPa – 0.69 MPa	Thakker et al.[3]
Zeolite 5A	Monolith with square channels	2.5 mm/min	0.05 MPa – 0.35 MPa	Thakker et al.[3]
CA/MIL-101/PEI	Monolith with square channels	0.2 mm/min	16 MPa - 17.5 MPa	Wang et al.[4]
Zeolite 13X	Gyroid monolith	1 mm/min	0.01 MPa – 0.24 MPa	Jivrakh et al.[5]

*Shear rate is used since the dimension of the sorbent is unknown.

References

(1) Li, Z. L.; Zhou, Y. L.; Yan, W.; Luo, L.; Su, Z. Z.; Fan, M. Z.; Wang, S. R.; Zhao, W. G. Cost-Effective Monolithic Hierarchical Carbon Cryogels with Nitrogen Doping and High-Performance Mechanical Properties for CO_2 Capture. *ACS Appl Mater Interfaces* **2020**, *12* (19), 21748–21760.

(2) Nazir, G.; Rehman, A.; Park, S. J. Self-Activated, Urea Modified Microporous Carbon Cryogels for High-Performance CO_2 Capture and Separation. *Carbon N Y* **2022**, *192*, 14–29.

(3) Thakkar, H.; Eastman, S.; Hajari, A.; Rownaghi, A. A.; Knox, J. C.; Rezaei, F. 3D-Printed Zeolite Monoliths for CO_2 Removal from Enclosed Environments. *ACS Appl Mater Interfaces* **2016**, *8* (41), 27753–27761.

(4) Wang, Y.; Rim, G.; Song, M. G.; Holmes, H. E.; Jones, C. W.; Lively, R. P. Cold Temperature Direct Air CO_2 Capture with Amine-Loaded Metal-Organic Framework Monoliths. *ACS Appl Mater Interfaces* **2024**, *16* (1), 1404–1415.

(5) Bharat Jivrakh, K.; Mathai Varghese, A.; Ehrling, S.; Kuppireddy, S.; Polychronopoulou, K.; Abu Al-Rub, R. K.; Alamoodi, N.; Karanikolos, G. N. 3D-Printed Zeolite 13X Gyroid Monolith Adsorbents for CO_2 Capture. *Chemical Engineering Journal* **2024**, 154674.
